# Perinatal clients’ experiences of care during COVID-19 in the North West District, South Africa

**DOI:** 10.4102/hsag.v30i0.2888

**Published:** 2025-04-30

**Authors:** Tebogo J. Matladi, Sharon H. Maluleke-Ngomane, Wanda Jacobs

**Affiliations:** 1 Department of Nursing, Faculty of Health Sciences, University of Johannesburg, Johannesburg, South Africa

**Keywords:** perinatal services, COVID-19, pandemic, pregnant women, experiences

## Abstract

**Background:**

The coronavirus disease 2019 (COVID-19) pandemic affected perinatal care services globally, leading to World Health Organization’s (WHO) recommendation for six in-person and two virtual antenatal visits to reduce travel and antenatal visits. Changes were made to reduce infection risk, with online consultations becoming a popular interim measure.

**Aim:**

The aim was to understand perinatal clients’ experiences of perinatal care during the COVID-19 pandemic.

**Setting:**

The study was conducted in five facilities of Bojanala district in the North West province, South Africa.

**Methods:**

The study followed a qualitative, exploratory, contextual design using semi-structured, face-to-face interviews. Purposeful sampling was used to select 10 participants meeting the inclusion criteria, who were interviewed until data saturation was reached. Data were organised into themes using thematic analysis. Ethical considerations and measures of trustworthiness were adhered to.

**Results:**

Three themes emerged altered routine, staff attitude and isolation during delivery. The nine sub-themes were pre-screening, entering the facility in turns, infection prevention and control, very slow queues, discontent about wearing masks, nurses’ fear of infection, staying longer than 5 h in the queue only to receive less than 10 min of service, no visitors including spouses allowed for emotional support, and compulsory COVID-19 test.

**Conclusion:**

South Africa should revisit policies and guidelines to support pregnant women during the difficult phases of unforeseen pandemics.

**Contribution:**

Multidisciplinary involvement in the psychological support of antenatal clients and nurses during any pandemic is crucial to ensure mental well-being.

## Introduction

### Background

Perinatal clients need to attend perinatal health services to ensure safe motherhood through early problem identification. Perinatal care is defined as the health service provided by skilled health professionals to clients during pregnancy and delivery to ensure best health condition of both the mother and the unborn baby throughout the pregnancy and during the delivery period (World Health Organization [WHO] 2016). According to the WHO, perinatal care is an important service delivery for monitoring, supporting and managing pregnant women and unborn children, to prepare for childbirth and to prevent any complications before and during the delivery (Geltore & Anore 2021).

Perinatal care services can be provided by both private and public primary health care sector. All government healthcare facilities offer free basic antenatal care (BANC) services and safe delivery. While most women cannot afford the full cost of services in the private sector, some still seek perinatal services in the private sector (Ebonwu et al. 2018). Perinatal care services were affected by the outbreak of the coronavirus disease 2019 (COVID-19) pandemic, as was the case with all health services (Ashokka et al., 2020). During the COVID-19 pandemic, WHO recommended six in-person visits and two virtual visits (3rd and 4th visits) to reduce the number of times the patient needs to travel and attend antenatal services (Uwambaye et al. 2020). In South Africa, perinatal care services were sustained throughout the initial two waves of COVID-19; however, there was a noticeable decrease in the utilisation of reproductive health services (Pattinson et al. 2020).

Changes were made to perinatal care in Europe to reduce the COVID-19 infection risk for pregnant women and staff, and online consultations were acceptable and valued by women as an interim measure (Coxon et al. 2020). Furthermore, face-to-face perinatal education programmes were discontinued across the board for women who had complex health conditions (NDoH 2020). In Southwest Ethiopia, since the COVID-19 pandemic, average weekly perinatal care visits have decreased by 16.1% (Peahl et al. 2019). During the COVID-19 pandemic, 13 161 pregnant women had their first antenatal visit before 20 weeks in the first quarter of 2019 in South Africa. In the fourth quarter of 2020, only 10 967 pregnant women visited the antenatal clinic for the first time before 20 weeks gestation, indicating a decrease of 17.0% (Pattinson et al. 2020). The attendance at antenatal clinics by pregnant women in the North West province decreased by about 8.77% during the pandemic (Pattinson et al. 2020). During the pandemic, perinatal clients were scared to attend antenatal care services because they feared infection.

In the Bojanala district in North West, perinatal clients were confronted with different obstacles in attending antenatal clinics during COVID-19, including long waiting periods because of COVID-19 protocols and fear of infection because some nurses did not put on their masks sometimes during screening. The researcher served as a primary health specialist at a facility within the Bojanala district, and identified a necessity to undertake this study, thereby providing an opportunity for psychosocial healing to the perinatal clients by affording them an opportunity to reflect on and share their experiences. Related studies have been conducted focussing mainly on the experiences of perinatal clients, but not in the context of Bojanala district; hence, the need for the study. The primary healthcare services statistics indicated that during the COVID-19 pandemic, the number of pregnant women who attended antenatal services decreased by 20% (National Department of Health 2022). This study seeks to understand the COVID-19-related experiences of perinatal clients during the COVID-19 pandemic.

## Significance of the study

The study aims to raise awareness among policymakers on the experiences of perinatal clients during a pandemic. Furthermore, to proactively make provisions for the improvement of perinatal care services during crises based on the research findings and enhance the resilience of the healthcare system. The study informs evidence-based policy and planning to promote a patient-centred care approach and strengthen infection prevention and control in a similar context. By investigating COVID-19-related disruptions to perinatal services in the Bojanala district, the study can contribute to strengthening healthcare systems and improving maternal and neonatal health outcomes in similar settings of South Africa and beyond.

## Research methods and design

### Design

This study adopted a qualitative, descriptive and contextual design in exploring COVID-19-related experiences of perinatal clients receiving services in Bojanala district, South Africa. Face-to-face unstructured interviews were conducted to gain in-depth knowledge of COVID-19-related challenges that affected perinatal clients in primary health care (PHC) facilities in Bojanala. Furthermore, observing, probing and analysing of non-verbal communication cues from 10 participants were done to ensure that the captured data represents the participants’ reactions and responses.

### Study setting

The study was conducted in five health facilities of the Bojanala district in the North West province, situated in the Northwestern part of South Africa, forming a boundary with Botswana on the South and Limpopo on the North. Bojanala district serves a population of 1 624 144 throughout its 125 healthcare facilities. Only 27 of the 125 healthcare facilities in the district are set up as community healthcare centres and offer 24-h services. Furthermore, 674 service sites are served by a total of 19 mobile clinics located around the five sub-districts: Moses Kotane, Rustenburg, Moretele, Kgetleng and Madibeng. This study was conducted in the Rustenburg sub-district, which has 23 health facilities with an average of 8 professional nurses in each facility. Five PHC facilities with midwife obstetric units (MOU) were selected purposely to render antenatal service as well as delivery and post-natal services.

### Population and sampling

The researcher recruited potential participants from the five PHC facilities who met the inclusion criteria. Participants attending perinatal care (antenatal and delivery services) at the selected community health care (CHC) were of interest in this study. As different perinatal clients attend perinatal care every day, they were briefly informed about the study and asked if they were willing to participate. Participants who agreed to be part of the study were asked to exchange their contact details with the researcher confidentially. The time, date and venue convenient to the participant were set for the interview. In this study, participants were conveniently sampled. The inclusion criteria were the participants between 18 and 49 years, who were low risk, and who were able to speak the local language as well as English. All high-risk clients and those who were below 18 years of age were not included.

### Data collection

The researcher made appointments with facility managers after receiving ethical clearance to request permission to conduct the study. In each appointment, the operational manager was resourceful in assisting with the process of accessing participants and sampling those who met the inclusion criteria. An information leaflet was read and explained in detail by the researcher to each participant in a face-to-face meeting to make an informed decision to participate or not. Those who volunteered to participate willingly signed a written informed consent form. Data were collected through semi-structured face-to-face interviews utilising an interview guide (Table 1) to allow the participants to express their experience in attending antenatal care services and delivery services during COVID-19. The interview guide played a crucial role, as it was developed based on the research objectives that outlined the research question, aims and objectives of this study. In addition, the methodological design was considered to ensure that the interview guide was in alignment with the overall research methods. A private room at the facility was used to conduct discussions to avoid external disturbance. The supervisors with expertise in qualitative research offered valuable guidance to the researcher regarding the conduct of interviews. In addition, the researcher utilised her communication skills acquired during her training in psychiatric nursing to effectively capture both verbal and non-verbal responses. Data collection was achieved through audio recordings and field notes to enhance the reliability of the findings. The pilot study was conducted with perinatal clients coming for antenatal care and delivery services in non-selected facilities to enhance the confirmability and credibility of the interview guide. The interviews was commenced in July 2023 and concluded in February 2024. Each interview lasted approximately 45 min–60 min.

**TABLE 1:** Interview guide.

Main question	Follow-up questions
The central question posed to the participants was, ‘How did you cope with attending perinatal services during the COVID-19 pandemic?’	How did you feel when you had to sit in the queue during COVID-19? How did you feel about the other antenatal clients that were in the queue with you? How did you cope with the other patients who came because they were sick? How long have you had to wait as the facility was sanitised after one patient tested COVID-19 positive? How did you feel delivering your baby with a mask? How did you feel about sharing a delivery space with other patients?

COVID-19, coronavirus disease 2019.

All interviews were conducted in the Setswana local vernacular language based on the preferences of the participants, translated into English, and checked by different language experts for accuracy. Data were collected using audio recorder and field notes to aid verbatim transcription. The participants were encouraged to elaborate on their challenges in attending perinatal care during COVID-19 through probing and follow-up questions. Data were collected until there was no new information generated at the 10th participant, which made the researcher conclude that saturation was reached. The central question posed to the participants was, ‘How was it for you to attend ANC services and delivery during this COVID-19 pandemic?’ Three phases of data collection were followed, which are: introduction phase, working phase and termination phase. In the interviews, the participants were probed to provide more information by asking questions such as: ‘Tell me more’. Further more the researcher applied techniques like listening actively, clarification, silence such as asking ‘mmm nodding head’. The termination phase provided a closing question: ‘Is there anything else that you need to share?’

### Data analysis

The researcher listened to the recordings several times to ensure familiarity with and to develop a deeper understanding of the data. After the last participant, which was informed by data saturation, the researcher transcribed all recorded interviews and sent them to an independent coder for coding. The themes and sub-themes that emerged from the interviews were carefully grouped and subsequently coded, categorised and clustered using thematic analysis (Braun & Clarke 2024). One main theme with three themes emerged, and an inductive process was used to derive sub-themes from the main themes. As coding occurred, the themes and sub-themes were linked to one another.

### Measures of trustworthiness

Trustworthiness was ensured using the four criteria of credibility, transferability, dependability and confirmability. Lincoln and Guba (1985) defined credibility as a measure of the truth of the data in the study to collect data and report findings. Credibility was established through triangulating sources, peer debriefing, member checking, persistent observation and participation in extended interactions with participants and the data, guaranteeing referential adequacy and preserving structural coherence (Stahl & King 2020). Dependability is the consistency and reliability of the study results (Polit & Beck 2021). Dependability was ensured through the dense description of the research methodology and context of the study. Transferability is the ability to transfer research findings from one context to another (Leavy 2022). Transferability was ensured by giving a thorough explanation of the target population, a thorough explanation of the research process, a designated sample and rich and comprehensive details on the study’s conclusions (Haq et al. 2023; Lincoln & Guba 1985). Confirmability was established through re-reading the transcripts, listening to the voice recordings and confirmation the supervisors and independant coder as well as providing direct quotes from participants (Haq et al. 2023; Lincoln & Guba 1985).

### Ethical considerations

Ethical clearance to conduct this study was obtained from the University of Johannesburg, by the Research Committee (REC 241112-035)(Clearance no.: REC-1299-2021). The participants were informed that their participation was voluntary and anonymous, and they could withdraw from the study at any time. The participants gave their informed written consent before the interviews and were assured of the confidentiality of their responses. Measures were taken to ensure confidentiality; a private room at the facility was utilised to conduct the interviews as it was convenient for the participants. The only people who had access to the recording were the supervisors, the researcher and the independent coder. The audio recorder and the transcribed data will be kept in an encrypted file for 5 years after the publication of the research output.

## Results

The demographic data of the participants indicating their age, relationship status, home language, parity (total number of children) and employment status are depicted in Table 2. To maintain confidentiality and anonymity, the participants were allocated a code that is from P1 to P10. The demographic data of 10 participants are indicated. Out of the 10 participants, only 1 was younger than 20 years; 5 were between the age of 20 and 29 years, and 4 were 30 years and older. Out of the 10 participants, 4 were married, and the rest were single. Two participants were employed, and eight were not working. Only one participant was Venda speaking, all the others were Tswana speaking. The interviews were conducted using the local vernacular language, which was Setswana. Tshi-Venda was the mother tongue of one participant, but the interview was conducted in Setswana and translated to English, because the participant was able to communicate without problems in Setswana. Participants 1, 2 and 9 did their interviews in English.

**TABLE 2:** Demographic data.

Participant	Age (years)	Relationship status	Home language	Parity	Employment status
P1	27	Single	Setswana	03	Unemployed
P2	30	Single	Setswana	01	Employed
P3	29	Single	Setswana	01	Unemployed
P4	18	Single	Setswana	01	Unemployed
P5	33	Married	Setswana	02	Unemployed
P6	37	Married	Setswana	02	Unemployed
P7	27	Single	Setswana	03	Unemployed
P8	34	Married	Setswana	03	Unemployed
P9	26	Married	Tshi-Venda	02	Employed
P10	21	Single	Setswana	01	Unemployed

Three themes and nine sub-themes emerged from the study (Table 3), which are:

- *Theme 1*: Altered clinic routine
- *Theme 2*: Staff attitudes
- *Theme 3*: Isolation during delivery

**TABLE 3:** Emerging main themes and sub-themes from participants’ experiences at healthcare centres while seeking care during COVID-19.

Themes	Sub-themes
Altered clinic routine	Pre-screening Entering the facility in turns Infection prevention and control Very slow queues Discontent with having to wear masks
Staff attitudes	Nurses fear to be infected Staying in the queue for more than 5 h only to receive a less than 10 min service
Isolation during delivery	No visitors, including spouses, were allowed for emotional support Compulsory COVID-19 test

COVID-19, coronavirus disease 2019; min. minutes, h, hours.

### Participants’ experiences at healthcare centres while seeking care during COVID-19

During COVID-19, there were various challenges that the participants faced during the antenatal care visits related to the changes in facility routine and protocols that were put in place to prevent infection. In the CHCs, there were new protocols to follow before entering the facility yard, such as screening for COVID-19. There were interruptions in the routine; for example, if there were cases of positive COVID-19, they needed to clean and disinfect. The participants reported that they had to queue for long hours and were attended to after a long time.

#### Theme 1: Altered routine in the service delivery

Since the pandemic, participants stated that there were changes in the service delivery at the facility and some of the changes were difficult to adapt to. Participants reported that they had to be screened for COVID-19 before entering the facility; hence, they had to stand in queues longer. It resulted in them spending the whole day at the clinic. Only after the participants were screened were they allowed to enter the clinic and be assisted in small groupings, extending the waiting time. This altered routine in the service delivery contributed to follow-up care fatigue.

**Sub-theme: Pre-screening:** The participants narrated that it was not possible to enter the facility before they were screened for COVID-19, and they did not like the instrument that was used to poke inside of the nose, causing bleeding, which happened every month when they came for services:

‘*Akere ga go kgonagale gore o ka tsena mo clinic o sa screeniwa*. [Isn’t it, it was not possible to get inside the clinic without being screened first].’ (Participant 4, 18 years old, unemployed)

‘*So ne go se monate cause ntho ele ne elematsa ele ba e kgotlhella ko deep ebe ke tswa madi a mantsi kgwedi le kgwedi fela ga keya clinic ya boimana*. [I was not happy with the thing they used to test us; they inserted it deeper inside the nose, and I was bleeding every month when attending antenatal.]’ (Participant 5, 33 years old, unemployed)

**Sub-theme: Entering the facility in turns:** Participants said that when they came for delivery, they had to wait 3 h for their turn to receive services, and they were not happy with the long waiting time:

‘*Gake yo go belega, ne go se Monate ke bile ka ema sebaka sa bo three hours ke me, ke immense gore ke tle nako ya ka ya go tsena klininking*. [When I went to give birth, I waited 3 hours waiting for my turn to enter the clinic.]’ (Participant 3, 29 years old, unemployed)

‘I was not happy at all, waiting for a long time for others to come out so that I could enter.’ (Participant 6, 37 years old, unemployed)

**Sub-theme: Infection prevention and control:** Some participants said the sisters at the clinic were seen not changing gloves between patients, and that made the patients scared of cross-infection. Some stated that the sisters did not put on their masks properly; they put them under their chin while they continued to talk:

‘*Akere bo sister ne vasa chichi di gloves ha among a while, ne ri tshaba go fillets* [Is in it that sisters did not change their gloves in between patients and we were scared to be cross infected.]’ (Participant 5, 33 years old, unemployed)

‘*Dimaski tsa bo sister ne di dula tlase ga seledu ge ba lapile, ene va tswela pele go bowa le rena*.’ [Sisters were putting their masks below their chin when they were tired; meanwhile, they continued to talk to us.]’ (Participant 4, 18 years old, unemployed)

‘Ehh, the air that I’m breathing, I feel like, is just the same as if I’m not wearing anything, so I have to put on a mask, at least two, to feel safe. Yeah, and I forgot, like is not, we were not only queuing two lines, there were three ‘cause we also have to do the testing for the HIV testing we also had to queue.’ (Participant 9, 26 years old, unemployed)

**Sub-theme: Very slow queues:** Participants said that the lines were too long and a social distance of at least 1.5 m from each other had to be maintained. Some participants stated that they would spend much time moving from one place to the other as they were called in few groups to enter. This was a challenge for them because they live far from the clinic:

‘*Di line tsa ko health centre ne dile ditelle and gote o nne le 1.5 metre* [At the health centre the lines were long and we had to keep a social distance of 1.5 metres from each other.]’ (Participant 3, 29 years old, unemployed)

‘You would spend a lot of time in the clinic, because they would take us in line like small group, 5–6, and they teach us; then they take 5–6 and teach them, you end up, uhm! spending the whole day in the clinic when you didn’t even plan to, mind you, I live very far from the clinic. So that was one of the challenges spending the whole day at the clinic during COVID.’ (Participant 1, 27 years old, unemployed)

**Sub-theme: Discontent with having to wear masks:** Participants verbalised discontent with having to wear masks while they are heavily pregnant. It made it difficult for them to breathe, and sometimes they ran out of breath. Furthermore, even when they were talking, they could not hear each other properly because they were sitting far from each other:

‘Ah, and you know, the issue with the mask, *Ijo*! with when you are heavily pregnant having to wear a mask, it’s kind of like difficult for you to breathe well … like you, you in most of the time, you run out of the breath.’ (Participant 1, 27 years, unemployed)

‘*Wa hupelwa and o tla be o sa ikutlwe sentle ka mask oo*. [You suffocate, and we could not hear ourself clearly with that mask.]’ (Participant 3, 29 years old, unemployed)

‘*Yanong jana ne lesa kgotlhagane o mongwe a nnela kwa, kafa di mask di re hupetsa*. [Now you are not supposed to sit together, and then the mask was suffocating us.]’ (Participant 6, 37 years old, unemployed)

‘Aee, wearing a mask was not easy because like you’ll feel like you are suffocating.’ (Participant 2, 30 years old, employed)

Participants stated that it was not comfortable for a pregnant woman to wear a mask as they are suffocating and that they were also not used to wearing masks every day.

Moreover, all pregnant or breastfeeding clients with unknown or previously negative HIV status should receive HIV testing on the same day that they present to the healthcare facility at every antenatal appointment, when in labour and 6 weeks after delivery (NDoH 2020). Participant 9 indicated that they had to queue for two lines because they had to have a COVID-19 test as well as HIV test done.

#### Theme 2: Staff attitudes

The participants stated that the staff did not allow them to extend their time at the clinical facility and that they were anxious to get the patients out of the system. The participants also stated that they felt that the staff feared contracting COVID-19 from their patients, which caused the staff to be less patient with them. Participants also reported that when there were COVID-19 cases in the clinic, services were stopped for hours or a day for disinfecting the clinic, and they had to leave and come back the next day.

**Sub-theme: Staying longer than 5 h only to receive a less than 10 min service:** Participants related that if there was one COVID-19-infected patient before they came, they had to wait while the clinic staff disinfected the clinic, which took long. The participants also stated that it took very long to arrive at the consultation room; but when they ultimately arrived, the nurses attended them hurriedly:

‘*Ga o tsena ko kliniking nako e nngwe gate go tswetswe*’ [When you arrive at the clinic some other times, they are closed.]’ (Participant 3, 29 years old, unemployed)

‘After the person who is infected by COVID they had to clean first.’ (Participant 4, 18 years old, unemployed)

‘*Ba satle gotla gore thusa ka nako, mara ga sere fehla go bone a bas ipha nako le rena*. [They didn’t attend to us in time, but when we ultimately arrived at the consultation rooms, they didn’t even give themselves time for us.]’ (Participant 3, 29 years old, unemployed)

**Sub-theme: Nurses feared to be infected:** Participants stated that sometimes the nurses at the facility treated them like they do not want to touch them. Another participant said that nurses thought that the patients are bringing COVID-19 to the clinic; hence, their moods change. Patients were not allowed to eat inside the clinic, and the nurses shouted at them and instructed them to wear their masks:

‘*Ne ba tshaba gore tshwara like bane ba re treat like bare nyonya nako engwe*. [They treated us like they don’t want to touch us sometimes.]’ (Participant 5, 33 years old, unemployed)

‘*Ne okase kgone goja ole mo jarateng ya clinic ne o tsie go o mangwa goterwala mask*. [It wasn’t allowed to eat inside the clinic yard, they would shout at you: “wear your mask.”]’ (Participant 5, 33 years old, unemployed)

‘*Bo sister le bone are yang ba phela ka letshogo la gore yang le rona rental ka covid retro go ba the kore mood o change one at all*. [Sisters are always scared of us, they think we bring COVID to them, the mood changed.]’ (Participant 7, 27 years old, unemployed)

The nurses were also afraid of being infected with COVID-19, but their behaviour and attitude towards the patients were not justified. Participants reported that the nurses made them feel like they had COVID-19 during the attendance of antenatal and natal services. Patients had to wait for a long time in queues; but when they arrived at the consultation room, nurses did not spend quality time with the patients (Walle et al. 2024). During the COVID-19 pandemic, the situation was further exacerbated by global shortages in personal protective equipment (PPE), increasing the risk to healthcare workers and patients (Phiri et al. 2021).

#### Theme 3: Isolation during delivery

Participants reported that their relatives and spouses were not allowed to assist and visit them when giving birth to avoid the spread of COVID-19. The relatives were turned at the gate and were asked to hand over the patients’ belongings or the items they had brought to security personnel who then distributed them to the patients.

**Sub-theme: No visitors allowed:** Participants said that their visitors were not allowed; if they brought something for them, it was handed to security personnel and they brought it to them. They further verbalised that their partners, who were supposed to give them emotional support, were not allowed to come because it is not allowed to have many people in the ward at once. Another participant said that even the people accompanying them had to turn at the gate because they were not allowed. These responses highlight a sense of isolation and frustration which the participants felt because no one from their families was allowed to see them:

‘Yah, so firstly, no visitors were allowed in the hospital.’ (Participant 1, 27 years old, unemployed)

‘*Ne ba sa tsene ne banna ko ntle kwa le ge ne ba go tsiseditse dipanana ne ba bollele ma security gore ba I set mang ko ward even*. [They {*family*} couldn’t enter; they were staying outside when they bring bananas; they tell the security guard to give you in which ward.]’ (Participant 6, 27 years, unemployed)

‘*Se sengwe gape ke gore aii batho ba rona balekane gaba sa tlhole ba kgona go tsamaya le rona ka gore ga o tsena ka fo go sister ka fale, sister o batla o tsene ole one fela ka gore ga la tshwanela go nna bantsi mo rumung gore room enyee le gore le tshwanetse gore le social distance akere sister*. [Another thing is that sister, hayi,! our partner is no longer accompanying us because they are no longer allowed to; the sister wants only you because you are not allowed to be many in the ward, and you have to do social distancing, sister.]’ (Participant 7, 37 years old, unemployed)

‘*Le batho ba neng o tsamaya le bone ba goisa hospital ne ba fella ko ntle ba baya dilo kontle*. [The people who accompanied you to the hospital were also not allowed to enter the hospital; they left your bags outside.]’ (Participant 3, 29 years old, unemployed)

**Sub-theme: Compulsory COVID-19 test:** Participants reported that every client who came to the facility needed to be tested for COVID-19, and only after all the tests had been done and had a negative status could they proceed inside the clinic. When they tested positive, they were sent home:

‘*Eiii ne ke sa I kutlwe sentle mara gonne ne ere gare tsena ebe bare test COVID gore amme re siame*. [Eii, I was not ok with it, but they tested us COVID before to check if we were ok.]’ (Participant 6, 27 years old, unemployed)

‘*Ga ona le disayini tsa corona, ne o dula kontle ba go teste pele, ene ga testi ile positive, oya sepetlele. Mara ha di li negative o romelwa gae*. [When you have signs of COVID-19 you stay outside, then go to the hospital, and if results come out positive, they tell you to go home.]’ (Participant 4, 18 years old, unemployed)

## Discussion

The aim of the study is to explore and describe the experiences of perinatal clients receiving care during the COVID-19 pandemic. A literature review was conducted to conceptualise the study. Three themes and nine sub-themes emerged from the study. Perinatal clients confirmed that they were stressed by the change of routine in the process of antenatal care in the facilities during the COVID-19 pandemic because they had to be screened first before they entered the facility, which perpetuated long queues taking them for more than 5 h to arrive to the consultation room. The findings of this study are supported by Horstman (2024), who found that women in labour were stressed when the facility routine changed because of health security issues. In this study, participants identified challenges related to attendance of perinatal care services during COVID-19. Despite challenges that were faced by pregnant women, maternity unit services had to operate to assist pregnant women. The findings show that pregnant women were not happy attending antenatal care services during COVID-19.

The research findings illustrate that pregnant women needed more attention and time during antenatal care from health care providers because stress levels were elevated. They were also in need of more psychosocial support because of the pandemic. These findings are supported by Miskeen (2024), whose study shows that fear of COVID-19, restrictions on resources, interrupted transportation and infection have been identified as major obstacles to perinatal participation during the pandemic. The qualitative results emphasised the ways in which security concerns affect healthcare access as well as the tactics of adaptation that have been used, such as telemedicine consultations and mobile antenatal clinics. Perinatal clients sensed they needed more and lacked support from their partners and families as they were not allowed to be accompanied.

Anxiety was further accentuated because the patients had to wait outside to be screened first for COVID-19, and if they tested positive, they were sent to the hospital unprepared. These results, supported by Mupambireyi (2024), show that patients were afraid they would not receive the finest care to help control infection or even receive better care. Based on the enormous death rates they were observing on news channels from affluent countries, women expected catastrophic consequences for themselves, their families and their communities. They minimised their travels and contacts as part of their preventive efforts; however, perinatal clients had to travel to facilities to receive care. At a time when people’s knowledge of the pandemic was limited, the myths and misconceptions that were spreading on social media platforms added to their worries (Thapa et al. 2024).

Another study supports this finding in that women who were isolated felt geographically abandoned, and that increased their anxiety during labour (Lognos et al. 2024). Furthermore, choices for pregnant women’s treatment pathways throughout the postpartum phase were restricted by their geographic remoteness. In addition to causing feelings of desertion, this resulted in higher follow-up expenses, particularly for those women seeking other types of pregnancy assistance. Partners wanting to participate in the pregnancy may have a sense of loss, and this affects fathers’ connection to their babies during pregnancy care (Atmuri et al. 2022). Pregnant women’s partners felt isolated because they were not allowed to come into the labour room because of changes made in maternity care during the pandemic (Nespolia et al. 2021).

A study of women giving birth in Italy during the pandemic found that women were having post-traumatic stress symptoms (Atmuri et al. 2022). The finding in the study was that pregnant women fear being in contact with COVID-19 or infecting their unborn babies, which can result in post-traumatic stress disorder. During the pandemic, healthcare organisations must ensure that all pregnant women are assessed for mental well-being.

Public transport was a challenge where participants had to wait for the slot before they could attend the clinic. There should be strategies on how to operate in terms of pandemics or crises; something needs to be done to overcome those challenges. This study might assist medical and psychological associates in a similar context in providing clinical recommendations for the support of pregnant women.

## Recommendations

The findings of this study highlighted the experiences of perinatal clients who attended antenatal care services during COVID-19. Through in-depth individual interviews, the study found that the participants had positive and negative experiences with the attendance of perinatal care during COVID-19. Recommendations have been made as per the findings for each theme and sub-themes.

### Recommendations for nursing practice

Coronavirus disease 2019 pandemic should be used as a benchmark for lessons learnt regarding perinatal care planning in all pandemics. The government must make sure that the facilities are well equipped with all the protective clothing and equipment and/or resources needed. Enough personnel should be employed and allocated for perinatal care to ensure that patients are not being neglected during a pandemic.

### Recommendations for nursing education, including in-service training

Ongoing in-service training must be conducted to ensure that all health professionals are aligned with the correct measures to minimise the spread of infectious diseases. Recent guidelines and standard operating procedures on the management of infectious diseases should be in place, and training should take place continuously in preparation for unexpected pandemics. The National Department of Health should allocate skilled personnel to visit healthcare establishments to mentor and support policy implementers and ensure quality of service delivery.

### Recommendations for nursing research

More research needs to be conducted to explore improvement strategies to support perinatal clients attending health services during a pandemic. Further research should be conducted to provide opportunities for spouses and relatives to share their experiences during COVID-19.

## Limitations of the study

The main limitation of this study is that the study only focussed on pregnant women attending antenatal care services and delivered after COVID-19.

## Conclusion

This study results met the study objectives and have answered the research question. From the interaction of the researcher and perinatal clients, the researcher was able to learn how perinatal clients needed to talk to someone after their experience of receiving services during COVID-19. This became an opportunity for debriefing from their emotional stress. Research findings were discussed, and the themes were merged with the literature review. The findings of this study showed that the pandemic interrupted attendance of perinatal care services among clients in the study area. The significance of nursing and implications for nursing education, nursing practice, nursing research and policies were discussed.
